# Environmental correlates of *Aedes aegypti* abundance in the West Valley region of San Bernardino County, California, USA, from 2017 to 2023: an ecological modeling study

**DOI:** 10.1186/s13071-025-06967-w

**Published:** 2025-08-18

**Authors:** Gaëlle T. Sehi, Solomon K. Birhanie, Jacob Hans, Michelle Q. Brown, Daniel M. Parker

**Affiliations:** 1 Population Health and Disease Prevention, Joe C. Wen School of Population and Public Health, University of California, Irvine, USA; 2 West Valley Mosquito and Vector Control District, Ontario, CA USA; 3 Epidemiology & Biostatistics, Joe C. Wen School of Population and Public Health, University of California, Irvine, USA

**Keywords:** *Aedes aegypti*, Vector ecology, Environmental monitoring, Earth observation, Geographic Information Systems, Mosquito control, Models

## Abstract

**Background:**

*Aedes* mosquitoes, particularly *Aedes aegypti* and *Ae. albopictus*, are major vectors of globally significant diseases such as dengue, Zika, and chikungunya. Since 2013, *Ae. aegypti* populations have rapidly expanded in California, making control efforts difficult due to their widespread, small-scale breeding sites and strong adaptation to urban environments.

**Methods:**

Remote sensing technologies, coupled with Geographic Information Systems (GIS), offer innovative solutions for mosquito surveillance and control. However, understanding the environmental drivers of mosquito abundance, particularly in California’s diverse ecological settings, remains an important gap. To address this gap, we analyzed *Ae. aegypti* abundance (2017 to 2023) in relation to environmental variables, such as temperature, precipitation, surface water, elevation, and built environment. We applied hotspot analysis to identify spatial clusters of high mosquito abundance and used a generalized additive model (GAM) with a negative binomial distribution to assess environmental and meteorological influences on mosquito counts.

**Results:**

Hotspot analyses revealed clusters of *Ae. aegypti* hotspots near residential areas. *Aedes aegypti* counts increased with higher surface water availability and temperature.

**Conclusions:**

Our study characterizes the spatial and temporal dynamics of *Ae. aegypti* mosquito abundance in the West Valley region of San Bernardino County from 2017 to 2023, shedding light on the influence of environmental factors and human activities on temporal trends. Our findings emphasize the critical role of temperature and water availability in shaping mosquito population dynamics, highlighting the need for proactive vector control strategies in response to environmental changes.

**Graphical Abstract:**

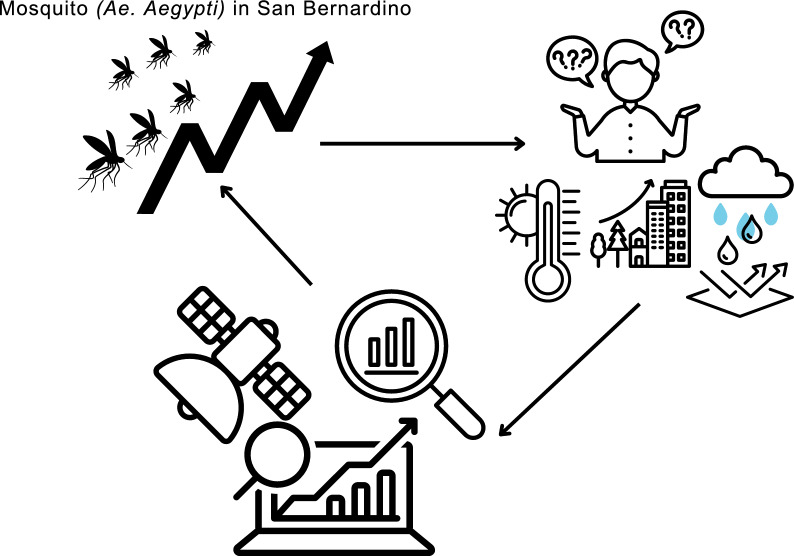

**Supplementary Information:**

The online version contains supplementary material available at 10.1186/s13071-025-06967-w.

## Background

*Aedes* mosquitoes (in particular *Aedes aegypti* and *Ae. albopictus*) are among the most important vectors of human disease globally [1]. They are the major vectors for several epidemiologically important viruses, including dengue, Zika, and chikungunya viruses. In most regions, the primary public health approach to controlling *Aedes*-borne diseases is vector-focused [2]. Understanding the ecology and spatio-temporal abundance of mosquito vectors can be important for targeting vector-focused interventions.

Environmental and meteorological factors have a major impact on the geographical distribution and abundance of mosquitoes. As ectotherms, ambient temperature has a non-linear association with mosquito activity, development, and population growth [3]. Temperatures that are too cold or too hot can negatively impact mosquito development and population growth [4–6]. Optimal temperatures can increase development and population growth [7].

The juvenile stages of mosquitoes are aquatic, meaning that water is crucial for mosquito habitation and population growth [8]. Precipitation is therefore frequently associated with increased mosquito populations, as it often leads to increased bodies of water that can serve as the necessary aquatic environment for the juvenile stages of mosquitoes. Other ecological and environmental factors are likewise important. *Aedes aegypti* is a container-breeding mosquito, preferring to lay eggs and spend its aquatic stages in human-made or naturally occurring containers that hold small bodies of water (e.g., cups, cans, old tires, bromeliads, tree holes) [9–17].

Given an optimal temperature and the presence of preferential bodies of water, *Ae. aegypti* can thrive in urban and suburban settings. While this mosquito species is known to have relatively short flight ranges (normally shorter than 200 m), it can disperse through human transportation routes, for example, through cargo ships [9]. Once this species has invaded a geographical region with suitable temperature and aquatic habitats, it is difficult to control, for a variety of reasons (e.g., many small cryptic habitats across a landscape can be difficult to find).

*Aedes aegypti* mosquitoes have been detected in California since 2013, and their range and abundance appear to be expanding [18–20]. By the end of 2023, the species had been detected in over 300 cities within 24 central and southern counties of the state [19]. Because of its vectorial capacity, strong adaptation to urban environments, and anthropophilic behavior [21], *Ae. aegypti* represents a significant risk for the transmission of *Aedes*-borne diseases. The detection of locally breeding populations of this vector has raised concerns among vector control agencies regarding the potential establishment of autochthonous transmission cycles in affected areas. Indeed, such local transmission has already occurred in Florida [22, 23], and in 2024, 18 locally acquired dengue cases were reported in southern California [24]. With travel-related *Aedes*-borne diseases increasing globally [25], the risk of local transmission remains concerning in California.

**Fig. 1:**
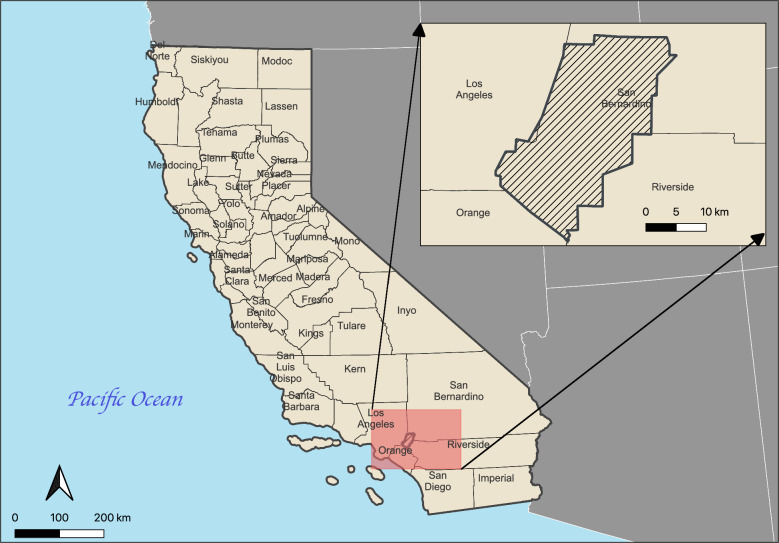
Study site location: West Valley Mosquito and Vector Control District, California, USA (black lines)

This study aims to examine granular spatial and temporal patterns of *Ae. aegypti* from 2017 to 2023 and to identify important environmental and meteorological factors associated with its abundance in the West Valley Mosquito and Vector Control District (WVMVCD) of San Bernardino County (Fig. 1). Since this species is of growing public health concern, an understanding of the factors that influence its geographical and temporal distribution is important for targeted mosquito control efforts.

## Methods

### Study area

The study was conducted in the WVMVCD of southwestern San Bernardino County, encompassing six cities—Chino, Chino Hills, Ontario, Upland, Montclair and Rancho Cucamonga—with a total estimated population of 600,000 (Fig. 1). The area is characterized by a Mediterranean climate, experiencing hot, dry summers and mild, wet winters [26]. Seasonal precipitation occurs predominantly between November and April, with occasional summer thunderstorms. Summer temperatures in the WVMVCD frequently surpass 38 °C, with occasional spikes exceeding 42 °C (Supplementary Figures S1 and S2). The native vegetation includes chaparral, oak woodlands, and riparian habitats, although urbanization has led to significant alterations in the landscape [27]. Furthermore, the region has experienced rapid population growth in recent decades, accompanied by extensive urban development, including residential, commercial, and industrial structures.

### Adult mosquito trapping

Mosquito surveillance was carried out by the WVMVCD, a special district responsible for vector surveillance and control in the region. Mosquito collections took place from January 2017 to December 2023 using BG-Sentinel traps (Biogents AG, Regensburg, Germany). BG-Sentinel traps are recognized for their effectiveness in monitoring *Ae. aegypti* across spatial and temporal trends [28]. Trapping sites were selected based on a combination of criteria: locations from which mosquito-related service requests had been recently received by the vector control district, and sites with a history of high *Aedes* abundance based on prior surveillance records. In addition, exploratory trapping was conducted in areas with no historical data to capture current mosquito dynamics. This targeted approach ensured optimal allocation of surveillance resources to areas of greatest public health concern and potential mosquito activity. Traps were deployed in shaded, wind-sheltered outdoor locations in close proximity to human habitations or areas of known mosquito activity, while avoiding direct sunlight and potential competing attractants. Each trap was equipped with a BG-Lure, a proprietary attractant that mimics human skin odors, placed in the designated lure compartment to enhance mosquito attraction. To further increase trap efficacy, an additional attractant, delivered via dry ice placed in insulated containers positioned adjacent to the trap, was included. Traps were powered by 12 V rechargeable batteries and operated continuously for a 24 h period. The following day, collection nets containing the captured mosquitoes were carefully removed from the traps, labeled, and transported in cool, insulated containers to preserve specimen integrity. In the laboratory, adult mosquitoes were knocked down by dry ice exposure and subsequently identified morphologically to species level under a microscope. Relevant data including Global Positioning System (GPS) coordinates were recorded for each sampling event to support interpretation of trap yields and spatio-temporal trends.

Trapping was systematically conducted throughout the district and set in residential areas. With the homeowner’s consent, traps were left overnight. Mosquitoes were collected the next morning and then counted and sorted by species. Each trap included a geographical location (latitude and longitude) and a timestamp indicating when it was set. Trap sites were strategically chosen based on historical mosquito hotspots, service requests from residents (e.g., complaints about mosquito biting), and an exploratory strategy to monitor mosquito activity across different areas of the district. All traps were brought to the WVMVCD Lab for mosquito counting and species identification by trained technicians. The district maintains records of adult mosquito counts, categorized by location and date, from 2017 to 2023.

The number of trapping sites varied from week to week depending on the number of *Aedes* mosquitoes collected at each location. Only traps that captured more than 20 *Ae. aegypti* individuals during the previous collection were eligible for redeployment, unless they were designated as routine sites. Additionally, mosquito-related service requests from residents informed trap placement, contributing to spatial variability across the study period. Notwithstanding these changes, eight routine sites were monitored weekly and consistently from 2017 to 2023 (see Supplementary Figure S10).

### Environmental and meteorological variables

Environmental and meteorological data were obtained through the Google Earth Engine (Table 1) from a variety of Earth observation data sources [29]. We extracted data on the built environment, elevation, precipitation, Normalized Difference Water Index (NDWI, a measure of surface water or moisture), and average ambient temperature—all chosen for their hypothesized or known impact on mosquito abundance (listed in detail in Table 1).

**Table 1:** Description of variables included in the final model

		Variable description	Source	Date range	Frequency
Outcome	Total *Aedes*	Counts of adult *Ae. aegypti* per trap	WVMVCD	Jan 1, 2017–Dec 31, 2023	Daily (aggregated to weekly)
Meteorological					
	Precipitation (mm)^a,b^	Log transformation of mean precipitation per trap	Copernicus Climate Change Service (C3S) ERA5-Land provides precipitation data with a resolution of 9 km [51]	Dec 1, 2016–Dec 31, 2023	Daily
	Average temperature (^o^C) ^a,b^	Mean maximum temperature per trap in Celsius (based on buffer around trap)	Copernicus Climate Change Service (C3S) ERA5-Land provides precipitation data with a resolution of 9 km [51]	Dec 1, 2016–Dec 31, 2023	Daily
Temporal	Day of the year	Day of the year on which the trap was checked (day 1 was the first day of the study year and day 365 was the last)	NA	Jan 1, 2017–Dec 31, 2023	Daily
	Year	Year of the study	NA	2017–2023	
Environmental					
	Surface water ^a,b^	Mean surface water index per trap (based on buffer around trap)	Sentinel-2 Multispectral Imager provides NDWI index value with a resolution of 30 m [52]	Dec 1, 2016–Dec 31, 2023	Every 5 days
	Elevation ^a^	Mean elevation from buffer around trap	NASA Shuttle Radar Topography Mission Global 1 arc second (SRTMGL1) product, with a resolution of approximately 30 m [53]	2023	Constant
	Built area^a^	Total number of pixels classified as “built” within the 150-m radius of each respective trap per year	Dynamic World provides near-real-time (NRT) land use/land cover (LULC) with a resolution of 10 m[54]	2017–2023	Yearly (taken from September)
	Longitude	Longitude of each trap	WVMVCD	Jan 1, 2015–Dec 31, 2023	Constant
	Latitude	Longitude of each trap	WVMVCD	Jan 1, 2015–Dec 31, 2023	Constant

*ERA5* European Centre for Medium-Range Weather Forecasts (ECMWF) European ReAnalysis, fifth generation; *WVMVCD* West Valley Mosquito and Vector Control District; *NA* not applicable

^a^Mean-centered and standardized

^b^14- and 28-day lags

These data were downloaded and merged into the trapping dataset based on location and trap date. Buffers 150 m in size were drawn around each of the trap locations, and environmental and meteorological variables were attributed to the traps based on these buffers. This buffer size was chosen based on the assumption that *Ae. aegypti* dispersal is generally limited to within 100 m of their breeding sites [30]. In our exploratory analyses, we also tested the use of 250-m buffers, based on a study from central California that suggested this species may have broader dispersal in nearby areas [31].

We extracted precipitation, surface water, and average temperature with 14- and 28-day lag periods from the mosquito collection date to account for the time needed for these environmental factors to influence mosquito development and population dynamics [32].

### Statistical analysis

#### Temporal patterns in trapped *Aedes*

We used descriptive statistics to summarize *Ae. aegypti* trap data from 2017 to 2023, including annual totals, means, standard deviations, medians, interquartile ranges, and the proportion of traps with zero mosquitoes. We investigated counts per trap by year to assess long-term trends in abundance, and by month across all years to detect seasonal patterns (results in Table 2 and Supplemental Table S1). These descriptive analyses helped contextualize environmental and spatial trends explored in later modeling.

**Table 2:** Summary statistics for trapped adult *Ae. aegypti* and traps by year

Year	Total *Ae. aegypti* caught	Total number of traps	Mean per trap	SD per trap	Median per trap	IQR per trap	Proportion of traps with no mosquitoes
2017	358	682	0.52	3.07	0	0	0.91
2018	661	750	0.88	2.59	0	0	0.76
2019	2954	772	3.83	7.28	1	5	0.44
2020	6404	898	7.13	11.24	2	9.75	0.30
2021	7953	991	8.03	15.77	3	11	0.31
2022	17,133	1009	16.98	41.97	6	19	0.25
2023	30,147	1538	19.60	32.43	7	24	0.23

*IQR* interquartile range, *SD* standard deviation

#### Spatial patterns in trapped *Aedes*

We mapped annual *Ae. aegypti* counts per trap from 2017 to 2023 to examine spatial patterns and changes in mosquito abundance over time. We then employed two main approaches to examine the potential spatial patterns of *Ae. aegypti* abundance from 2017 to 2023. First, we created spatial correlograms for each year to investigate how spatial autocorrelation in mosquito abundance changed with increasing distance between trap locations. These correlograms were constructed by calculating Moran’s *I* at increasing distance intervals up to 3 km, using 500-m bins. To account for sampling effort variations, we used the mean weekly number of mosquitoes per trap location within each year for these calculations. This approach provided insights into the scale of spatial dependence and its annual variations.

Following this global assessment, we employed the Getis-Ord Gi* statistic to identify significant local clusters of high (hot spots) and low (cold spots) *Ae. aegypti* abundance. We applied a fixed-distance spatial weights matrix with a 500-m threshold, selected based on results from the correlogram analysis. Locations with statistically significant high positive *Z*-scores (> 1.96, *P* < 0.05) were identified as hot spots, while those with statistically significant low negative *Z*-scores (< −1.96, *P* < 0.05) were classified as cold spots.

To ensure robustness, we conducted a sensitivity analysis by repeating the correlogram analysis and Getis-Ord Gi* using 250-m bins/threshold, comparing results with our primary analysis (Supplementary Figures S4 and S5). We generated annual hotspot maps and created a combined visualization of annual spatial correlograms.

This multi-approach method allowed us to track changes in spatial clustering patterns over time. Our exploratory spatial analyses provided a foundation for investigations into environmental correlates of *Ae. aegypti* abundance.

#### Bivariate analysis between environmental and meteorological covariates and *Ae. aegypti* abundance

We hypothesized that the spatial and temporal distribution of *Ae. aegypti* in the WVMVCD would be positively associated with precipitation, surface water, and the built environment. We also hypothesized that higher ambient temperatures would be associated with higher mosquito abundance. To visualize potential associations between *Ae. aegypti* abundance and environmental factors, mosquito abundance data from traps were plotted against continuous environmental variables. This approach allowed for a preliminary examination of potential associations between each factor and *Ae. aegypti* abundance in our study area.

#### Generalized additive model for associations between environmental and meteorological covariates and *Ae. aegypti* abundance

We then used a negative binomial generalized additive model (GAM) to model counts of trapped *Ae. aegypti* mosquitoes, using the environmental variables as covariates in the model and accounting for trap location and time, for the duration of the 2017–2023 study period. The GAM allowed us to account for the complex and potentially non-linear associations between environmental variables and *Ae. aegypti* abundance through the use of smoothing spline functions [33].

To account for potential lagged effects (i.e., precipitation might have a delayed effect on mosquito abundance), we explored different lags (14 and 28 days) for the precipitation, surface water, and average temperature. We began by developing a model for all data from 2017 through 2023, with all covariates specified as thin plate regression spline functions. All covariates were centered on their means and standardized by their standard deviation to facilitate the interpretation and comparison of effect sizes.

Final variable selection and model specification was based on a combination of scientific hypotheses, model goodness-of-fit tests (deviance explained and the Akaike information criterion (AIC)] (Supplementary Tables S2 and S3), and interpretability—referring to the inclusion of variables with meaningful, biologically relevant associations that could inform vector control strategies. Through comparison of model fit using deviance explained and the AIC, we determined that a 14-day lag for these variables provided the best model fit. The final negative binomial GAM included the following variables: NDWI, precipitation, built environment, elevation, average temperature, geographical location, season, and year. It also incorporated interaction terms between season and average temperature, as the effect of temperature shifted over time.

The results of our regression analysis are presented as graphs showing the estimated smoothed spline functions for each predictor variable (Fig. 6), along with a summary table indicating statistical significance (Supplementary Table S2).

#### Software

All statistical analyses were performed using the R statistical software (version 4.3.3). The spatial autocorrelation analyses were run using the spdep and tmap packages. The GAM was created using the mgcv package. All maps were created using QGIS (version 3.34.9).

## Results

### Temporal distribution of trapped *Ae. aegypti* mosquitoes

The abundance of *Ae. aegypti* increased in this setting over the study period (2017–2023), even while accounting for increased surveillance and trapping. While only 358 adult *Ae. aegypti* were trapped in 2017, 2954 were trapped in 2019 and 30,147 were trapped in 2023. While trapping and surveillance increased from 682 traps set in 2017 to 1538 in 2023, the mean and median numbers of *Ae. aegypti* caught per trap night increased from 0.52 and 0 (mean and median, respectively) per trap in 2017 to 19.6 and 7 per trap (mean and median, respectively) in 2023. The proportion of traps set that did not catch any mosquitoes also decreased over this period, from 91% in 2017 to 23% in 2023 (Table 2).

There was a clear seasonal pattern in trapped adult *Ae. aegypti* mosquitoes as well (Supplementary Table S1). August, September, and October had the highest numbers of trapped mosquitoes (higher mean per trap, higher median per trap, and lower proportion of traps catching zero adult *Ae. aegypti*). This time period roughly corresponds to the local dry and hot season (Supplementary Figures S1 and S2).

### Spatial patterns in trapped *Ae. aegypti* mosquitoes

**Fig. 2:**
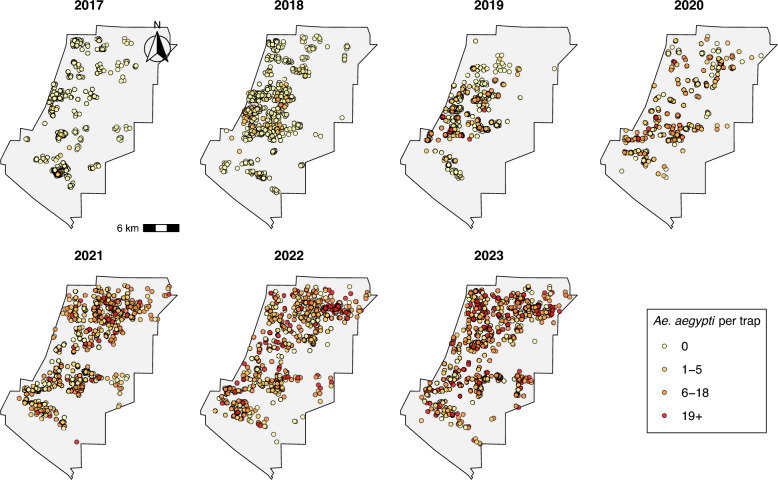
Spatial distribution of traps and adult *Ae. aegypti* counts, 2017–2023. Locations of BG-Sentinel traps, and relative numbers of adult *Ae. aegypti* caught in traps, from 2017 to 2023

The increase in abundance of trapped *Ae. aegypti* in the WVMVCD during the study period (2017–2023) was likewise evidenced in maps of trapped mosquitoes (Fig. 2). Although trapping and surveillance efforts expanded during this time, they do not fully explain the increase in abundance. The standard deviation in mosquito counts per trap increased from 3.07 in 2017 to 32.43 in 2023 (Table 2), indicating growing variability and suggesting that mosquito infestation became more spatially uneven, with the emergence of high-abundance hotspots alongside low-abundance areas.

**Fig. 3:**
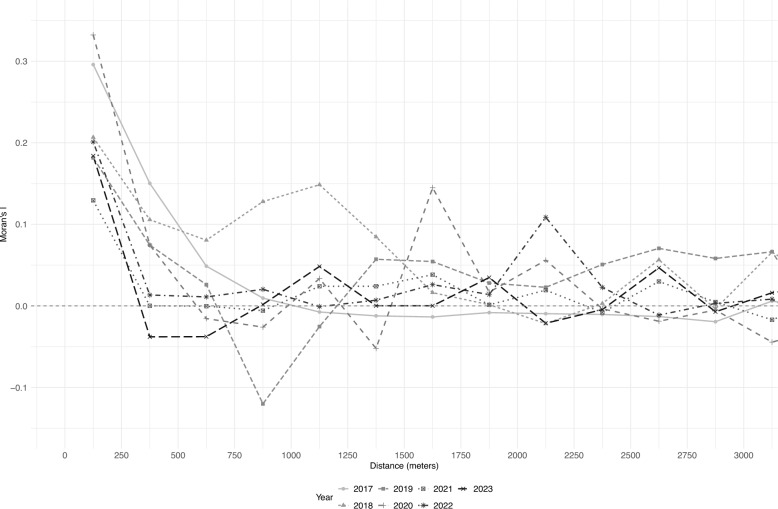
Spatial correlogram for *Aedes* mosquitoes (2017–2023). The spatial correlogram shows Moran’s *I* value at varying distance classes to assess spatial autocorrelation in *Aedes* mosquito abundance from 2017 to 2023. Positive Moran’s *I* values at shorter distances indicate the clustering of mosquito populations, with spatial dependence weakening at larger distances. This analysis informed the hotspot mapping shown in Fig. 4

Spatial correlograms for *Ae. aegypti* from 2017 through 2023 indicate positive spatial autocorrelation (measured using Moran’s *I* statistic) at relatively small distances (in particular < 500 m) (Fig. 3). All but one year (2018) showed positive clustering up to approximately 1 km, and four years had clustering at 500 m or less. No clear and consistent pattern of clustering was apparent at distances larger than 1 km.

**Fig. 4:**
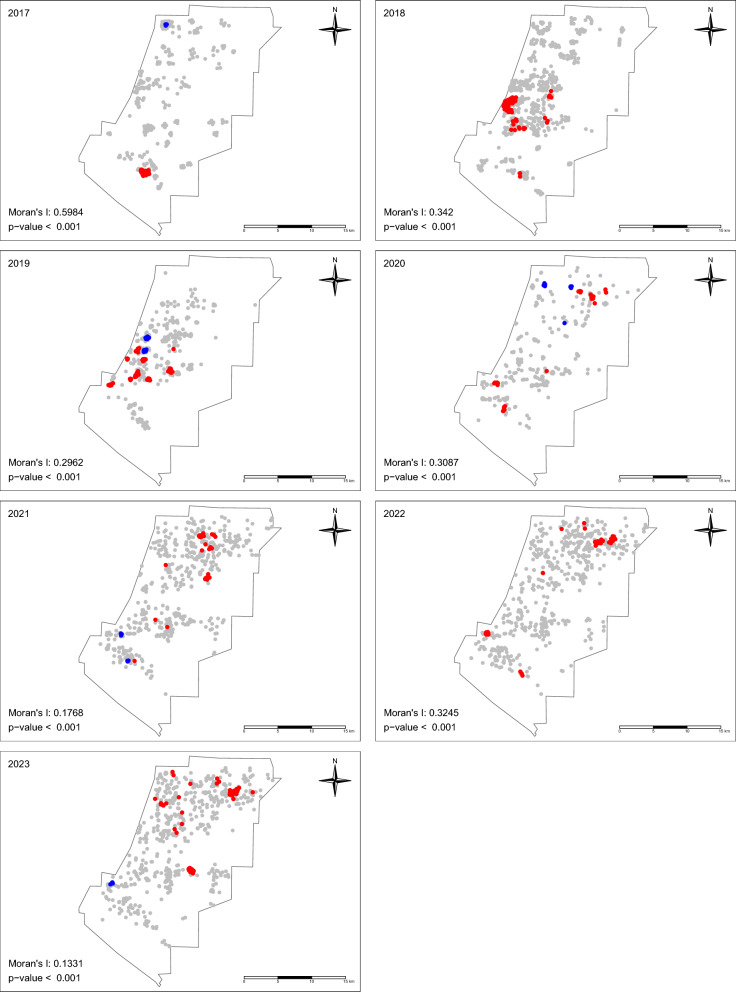
Hotspot analysis of *Ae. aegypti* abundance (2017–2023). The hotspot analysis shows year-to-year differences in the spatial distribution of *Ae. aegypti* abundance across the study area. Hot spots (red) represent areas with statistically significant high mosquito abundance, while cold spots (blue) indicate areas with statistically significant low mosquito abundance, both relative to the overall study area

Additionally, our spatial analysis using the Getis-Ord Gi* statistics with a 500-m bin revealed dynamic patterns in *Ae. aegypti* abundance from 2017 to 2023 (Fig. 4). In the early years, hot spots were concentrated in the south (i.e., near Chino Hills State Park and residential areas). Over time, these clusters shifted and new hot spots emerged in the western, central, and northern parts of the study area, encompassing diverse land use types including parks and residential and business zones. Notably, the Chino Hills State Park hotspot persisted for 2 years and then transitioned from a hot spot to a cold spot by 2019. While some hot spots showed temporal stability from 2020 to 2022, several became cold spots by 2023. Moran’s *I* values also declined during this period, from 0.59 in 2017 to 0.13 in 2023, reflecting a decrease in overall spatial autocorrelation.

### Correlations between meteorological and environmental variables and *Ae. aegypti* abundance

**Fig. 5:**
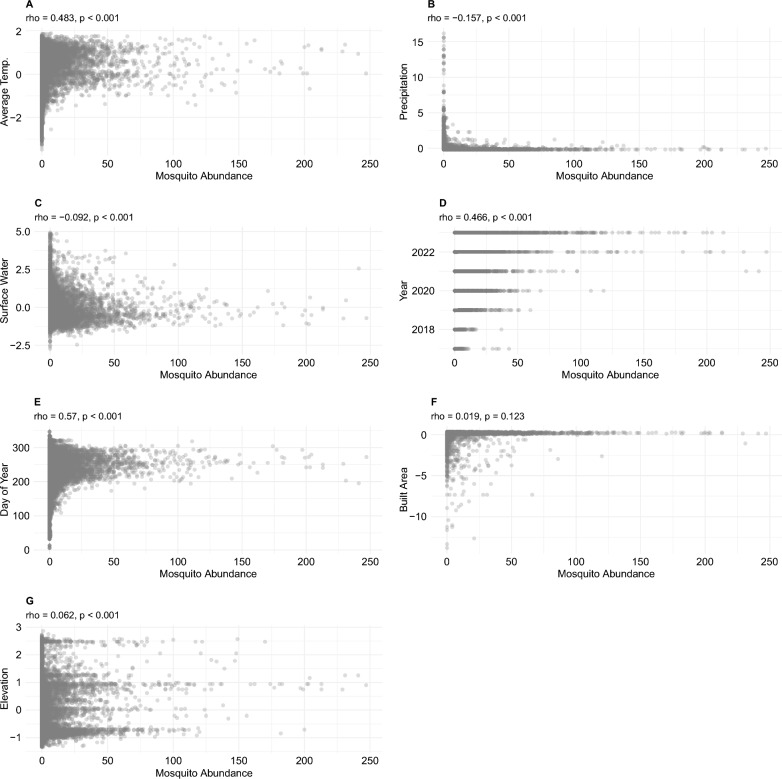
Bivariate analysis of environmental, meteorological, and temporal variables associated with mosquito abundance from 2017 to 2023. Scatterplots between *Ae. aegypti* abundance (*x*-axis on all plots) and environmental, meteorological, and temporal variables. Spearman correlation coefficients (rho) are shown on top of plots and indicate the relative strength and direction (positive or negative) of each association. **A** Average temperature; **B** precipitation; **C** Normalized Difference Water Index (NDWI); **D** year; **E** day of the year (DOY, representing seasonality); **F** built environment; **G** elevation (digital elevation model [DEM])

The bivariate analysis was conducted using Spearman’s rank correlation, which is appropriate for detecting monotonic relationships, including non-linear associations and non-normally distributed variables. This method indicated positive associations between temperature, seasonality, and recent years with higher mosquito abundance, while elevation showed a weaker positive association (Fig. 5). Precipitation displayed a complex pattern, and surface water and built area showed no clear relationships with mosquito abundance.

Temperature (Fig. 5A, rho = 0.20) showed a positive association with mosquito abundance, with higher values corresponding to higher average temperatures. This pattern was similar to the observed seasonality (Fig. 5E, rho = 0.261), where higher mosquito abundance clustered around late summer to early fall (around day 250). Temporal trends (Fig. 5D, rho = 0.258) were observed, with later years (2020–2022) correlating with higher abundance.

Precipitation (Fig. 5B, rho = −0.057) exhibited a complex relationship with abundance. Most observations showed consistently low values, while higher values were occasionally associated with outliers. NDWI (Fig. 5C, rho = −0.052) and built area (Fig. 5F, rho = −0.004) displayed minimal variation, showing no clear association with mosquito abundance in this context. Elevation (Fig. 5G, rho = 0.081) demonstrated a slight positive association with abundance, with higher mosquito abundance occurring at slightly lower elevations.

Although several correlations were statistically significant, their relatively low magnitude highlights the importance of using multivariable models to capture complex relationships among predictors.

### Results from the negative binomial GAM for environmental and meteorological correlates of *Ae. aegypti* abundance

**Fig. 6:**
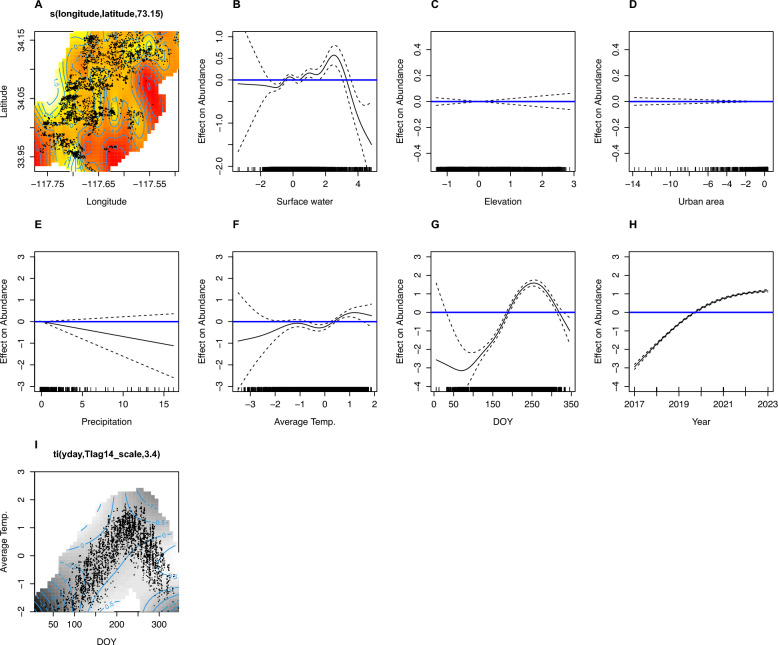
Spline function results from the generalized additive model (GAM) for *Ae. aegypti* abundance from 2017 to 2023. **A** Geographical coordinates; **B** Normalized Difference Water Index (NDWI); **C** elevation (digital elevation model [DEM]); **D** built environment; **E** precipitation; **F** average temperature; **G** day of the year (DOY, representing seasonality); **H** year; **I** interaction between day of year and average temperature. As our variables are centered on their means, the *x*-axis in these plots represents deviations from the average value of each predictor. The *y*-axis shows the relative effect on mosquito abundance, centered around zero. This allows for the interpretation of how deviations from the mean of each predictor are associated with changes in expected mosquito counts while holding other variables constant at their average values. Values where the spline and its confidence bands are above the blue line indicate statistical significance and a positive association with *Ae. aegypti* abundance, for the respective values of the covariate, indicated on the *x*-axis. Conversely, spline functions that reach below the blue line indicate values (along the *x*-axis) for that covariate that are associated with lower *Ae. aegypti* abundance. For the interaction spline (**A** for latitude and longitude, and **I** for temperature and day of year), the three-dimensional association is viewed as a contour plot instead. For both, lighter values indicate areas on the contour plot associated with higher counts of *Ae. aegypti* mosquitoes. See Supplemental Figures S7 and S8 for enlarged versions of plots (**A**) and (**I**)

The spline interaction term for latitude and longitude (Fig. 6A) is a representation of the association between location and abundance of *Ae. aegypti*, after accounting for the other covariates in the model. This map demonstrates the clustering of mosquito abundance in the south and central-west parts of the study area, as indicated by the bright white and yellow areas on the map.

Surface water (Fig. 6B) and average temperature (Fig. 6F) showed positive associations with *Ae. aegypti* abundance. There was a positive association with elevation at low to mean levels of the variable, but all higher-elevation areas showed no statistical significance (Fig. 6C—confidence bands on both sides of the 0 point on the *y*-axis beginning at roughly 0.5 standard deviations above mean elevation and extending to all higher elevations). The model showed no association between the built environment (“urban area” Fig. 6D) and mosquito abundance, or with precipitation and *Ae. aegypti* abundance (Fig. 6E).

A strong seasonal pattern was evident (Fig. 6G), with higher abundance occurring during August, September, and October of each year. Furthermore, the general increase in abundance over time (years) was apparent in the model (Fig. 6H), indicating an increase in *Ae. aegypti* abundance over the study period (2017–2023), even after accounting for the other covariates in the model.

Finally, the interaction between average temperature and day of the year (Fig. 6I) revealed that the effects of temperature on *Ae. aegypti* abundance varied throughout the year. In early spring (March–April), lower temperatures corresponded to lower mosquito counts. During the summer months (May–September), higher temperatures were associated with increased abundance. This relationship extended into early autumn, with elevated counts persisting for a short period after temperatures began to drop in October. Temperature ranges varied significantly across seasons, with lower temperatures during peak summer months (July and August) exceeding the highest temperatures observed during the coldest months (December and January).

We ran a sensitivity analysis using 28-day lags for the time-varying covariates (see Supplementary Table S3 and Figure S3). The model was largely the same, with the exception that the ambient temperature from 28 days before a trap date was not a significant predictor of mosquito abundance (whereas in the main model presented here, ambient temperature 14 days before trapping was a significant predictor) (Supplementary Table S2).

## Discussion

Our study on *Ae. aegypti* mosquito abundance from 2017 to 2023 in the WVMVCD (part of San Bernardino County) in southern California revealed several key findings. We observed a significant increase in *Ae. aegypti* abundance over the study period, from an average of 0.52 mosquitoes per trap in 2017 to an average of 19.60 mosquitoes in 2023, with yearly increases corresponding to summer months. We found that surface water, temperature, seasonality, and geographical location were predictors associated with mosquito abundance. Additionally, spatial clustering of high mosquito abundance was concentrated in the central and southern regions of the study area—though clusters did shift over time.

In our analysis, surface water was associated with *Ae. aegypti* abundance. Conversely, precipitation was not associated with abundance. The region is semi-arid, with very little precipitation in an average year. The lack of a clear relationship between precipitation and *Ae. aegypti* abundance could be explained by the ecology of this species. This mosquito often breeds in small artificial containers located around human dwellings, meaning seasonal patterns in population dynamics might be more influenced by variations in peridomestic water availability than changes in precipitation. Heavy rains could likewise have a negative effect on larval abundance, since intense rainfall may flush the larval habitat, thereby affecting the adult mosquito population [34–36]. We also note that in this setting, surface water does not come from precipitation alone, since patterns in surface water over time did not align with precipitation. We hypothesize that much of this surface water is from residential yard watering and agricultural irrigation, which increases during prolonged periods of the dry season without precipitation. While surface water availability may serve as a potential predictor of *Ae. aegypti* distribution, its influence is dependent on additional environmental and anthropogenic factors.

We found that average temperature was also an important factor with regard to the abundance of *Ae. aegypti* mosquitoes. In our model, average temperature significantly influences *Ae. aegypti* abundance, but it is important to note that seasonal factors, including photoperiod, could modulate the effect of temperature on mosquitoes. The interaction between temperature and season highlights the complexity of mosquito population dynamics, where optimal conditions depend on multiple environmental factors [37]. While warmer temperatures during peak season generally promote mosquito development, extreme heat can reduce habitat suitability. Longer daylight hours in summer enhance mosquito activity and reproduction, whereas shorter daylight hours in cooler months suppress it, even when temperatures remain favorable [38]. These findings emphasize the need to consider both temperature and seasonal cues in predicting mosquito populations to improve vector control strategies.

Geographical location plays a crucial role in *Ae. aegypti* abundance (Fig. 6A). We did not find a significant association between built area and mosquito abundance in our model, likely because most traps were placed in urban areas. However, using spatial analysis, we identified localized spatial clustering of *Ae. aegypti* abundance in various locations across the study area. For example, the spatial correlogram analysis showed that *Ae. aegypti* populations are highly localized, and the Getis-Ord Gi* analysis revealed spatio-temporal shifts in *Ae. aegypti* abundance, with initial hotspots concentrated in the southern part of the study area in 2017 gradually expanding and relocating to central and northern regions in subsequent years. This localized clustering aligns with the well-documented limited flight range of *Ae. aegypti*, which typically disperse only short distances from breeding sites—often less than 200 m—highlighting the importance of fine-scale spatial analysis in detecting meaningful patterns [39]. Our regression analyses suggest that some of these spatial and temporal patterns are driven by micro-environmental factors such as water availability, ambient temperature, and human activity [40].

Many of these hot spots were observed in residential areas and near public spaces such as parks and schools, likely due to the presence of suitable breeding sites and human hosts. Clusters of high mosquito abundance were also noted in proximity to commercial areas, including real estate developments, fire sprinkler system businesses, junk removal services, and major roads (e.g., Highways 210 and 110). Cold spots, while less frequent, were observed in some locations, including near a senior center. The distribution of both hot spots and cold spots varied throughout the study period, with some areas showing persistent patterns and others demonstrating changes over time. This shift in the spatial distribution was also reflected in a decrease in global spatial autocorrelation over time, as measured by Moran’s *I*, suggesting a transition from tightly clustered to more dispersed patterns of mosquito abundance across the district.

The spatial and temporal variability in *Ae. aegypti* abundance has important implications for vector control strategies. Persistent hotspots may require targeted, intensive interventions, while areas with fluctuating patterns suggest the need for adaptive, flexible approaches. Understanding these dynamics is integral to optimizing resource allocation in mosquito control efforts, whether through traditional methods, novel techniques like sterile insect release, or integrated vector management strategies. This knowledge can guide decision-makers in prioritizing high-risk areas, adjusting intervention timing and intensity, and potentially improving the cost-effectiveness of control measures. Ultimately, these insights contribute to more efficient and sustainable vector management. While our study does not assess mosquito infection or human case data, the characterization of spatial and temporal patterns of *Ae. aegypti* abundance provides a foundation for proactive vector surveillance, which is an essential component of public health preparedness in urban environments.

The spatial and temporal patterns we observed in *Ae. aegypti* abundance reflect the complex dynamics of this species’ expansion into new environments. *Aedes aegypti* has been expanding in the southwestern parts of the United States in recent years [41], a trend of particular concern given the large population in California, the relatively frequent travel-related *Aedes*-borne diseases, and the recent occurrence of locally transmitted dengue in the region [42]. This highlights the importance of proactive vector surveillance, even in the absence of widespread local transmission. The relationships that we observed, which differ somewhat from what is normally seen in tropical and subtropical regions of the world where the mosquito is endemic (and normally more common in the rainy season) [13, 43, 44], highlight potential challenges for public health and vector control agencies. Heightened surveillance that includes ecological research into the environmental, meteorological, and geographical correlates of *Ae. aegypti* abundance is important for understanding seasonal, inter-annual, and geographical patterns in abundance. As this species moves into new environments, identifying consistent environmental predictors of its presence and abundance may remain challenging until it reaches equilibrium with local environmental conditions.

This study has both limitations and strengths. Our assessment of mosquito abundance is limited to locations and time periods when trapping occurred (February to November). Over time, trapping efforts expanded geographically, coinciding with an increase in *Ae. aegypti* abundance. While our model accounted for trapping intensity, we were unable to assess abundance in areas and time periods without trapping. Additionally, trapping itself may influence *Ae. aegypti* abundance, as mosquitoes removed from the environment are not replaced immediately [28]. Moreover, the use of cluster trapping—deploying additional traps around high-count sites to identify potential mosquito sources—may have contributed to localized increases in mosquito abundance in certain instances. Overall, the observed reduction in the proportion of traps yielding zero *Ae. aegypti* is however most likely due to the spatial expansion of the mosquito population across the district. Over time, *Ae. aegypti* became more widespread and ubiquitous, as reflected by higher detection rates and a declining number of traps with no mosquito captures. Nonetheless, we believe the trapped mosquitoes provide a reliable representation of abundance in the surrounding environment. Our analyses incorporated Earth observation data, primarily derived from satellite imagery. These data can be affected by cloud cover, potentially leading to incomplete surface water measurements [45]. However, unlike tropical regions, where cloud cover can persist for weeks or months, this issue is relatively minor in this arid part of North America. There were relatively few periods during the study when Earth observation data were unavailable. We were also unable to directly incorporate photoperiod into the model, as daylight exposure varies by trap location and is not routinely measured; however, seasonal variation in daylight length is partially captured through the inclusion of day of year and temperature.

Despite these limitations, this study has several notable strengths. The use of Earth observation sensors (e.g., satellite systems) allowed for remote monitoring of environmental conditions at fine spatial and temporal resolutions across much of the study period [46]. Such high-resolution, long-term monitoring would have been cost-prohibitive and logistically challenging using only ground-based methods. As the availability and accessibility of Earth observation data continue to grow, their integration into vector-borne disease surveillance systems in this and other settings is expected to expand [47]. Additionally, the extensive surveillance data collected over a 7-year period represents a key strength of this study. The depth and breadth of the spatial and temporal dataset facilitated a robust analysis of mosquito distribution patterns, revealing both long-term trends and localized variations. Recognizing the value of predictive modeling in forecasting vector abundance, we are currently exploring advanced approaches—such as machine learning algorithms and spatio-temporal models—as part of our ongoing and future research efforts.

Finally, as the threat of *Aedes*-borne diseases in this region increases, vector control agencies have moved toward the implementation of different novel interventions [42]. In the WVMVCD, sterile insect release is currently being incorporated into vector control efforts [48, 49]. Recent work indicated a reduction in *Ae. aegypti* abundance by up to 65% following the optimization of integrated vector management strategies through adoption of the target sterile insect technique and In2Care Mosquito Stations in selected sites in the area in 2024 [50]. The model presented here, which accounts for environmental factors influencing *Ae. aegypti* abundance, can be adapted to assess the relative impact of this intervention while accounting for spatial and temporal variability.

## Conclusions

In conclusion, this study highlights a significant increase in *Ae. aegypti* abundance in the WVMVCD of San Bernardino County over the past 7 years, and highlights complex relationships between environmental, meteorological, and human-driven factors and their abundance. Our findings emphasize the importance of surface water—potentially driven by human activities such as garden irrigation—alongside temperature and seasonality as key predictors of mosquito abundance in this arid region. The integration of long-term surveillance data with satellite-derived environmental metrics provides a strong foundation for ongoing research and monitoring efforts, which will be important as the region is now incorporating innovative vector control approaches such as sterile insect release. Accurately modeling the relative impact of such interventions while accounting for geographical and environmental drivers of abundance will be essential. While our study does not assess mosquito infection or human case data, the characterization of spatial and temporal patterns of *Ae. aegypti* abundance provides a foundation for proactive vector surveillance, which is an essential component of public health preparedness in urban environments.

## Supplementary Information

Supplementary Material 1.

## Data Availability

Data supporting the main conclusions of this study are included in the manuscript.
