# Supplementary material for: Environmental correlates of Aedes aegypti abundance in the West Valley region of San Bernardino County, California, USA, from 2017 to 2023: an ecological modeling study
Source: Parasit Vectors. 2025 Aug 18;18:349. doi: 10.1186/s13071-025-06967-w (PMC12359924; doi:10.1186/s13071-025-06967-w)
Supplement: Supplementary file 1 — Supplementary Material 1. [file 13071_2025_6967_MOESM1_ESM.docx]

**Supplementary Material**

**Model Equation.**

Full Model

Total Aedes ∼ Surface water + Built environment + Elevation + Precipitation + Average Temperature +s(longitude,latitude) + s(Day of the year) + s(year) + ti(Day of the year* Average Temperature)

where s() indicates a spline function, ti() indicates tensor product interaction

**Figure S1**. **Example meteorological patterns during the study time from a weather station at Ontario International Airport, located near the study location.** The top panel indicates monthly high and low (max and min) ambient temperatures. The lower panel indicates total precipitation by month during the study period.


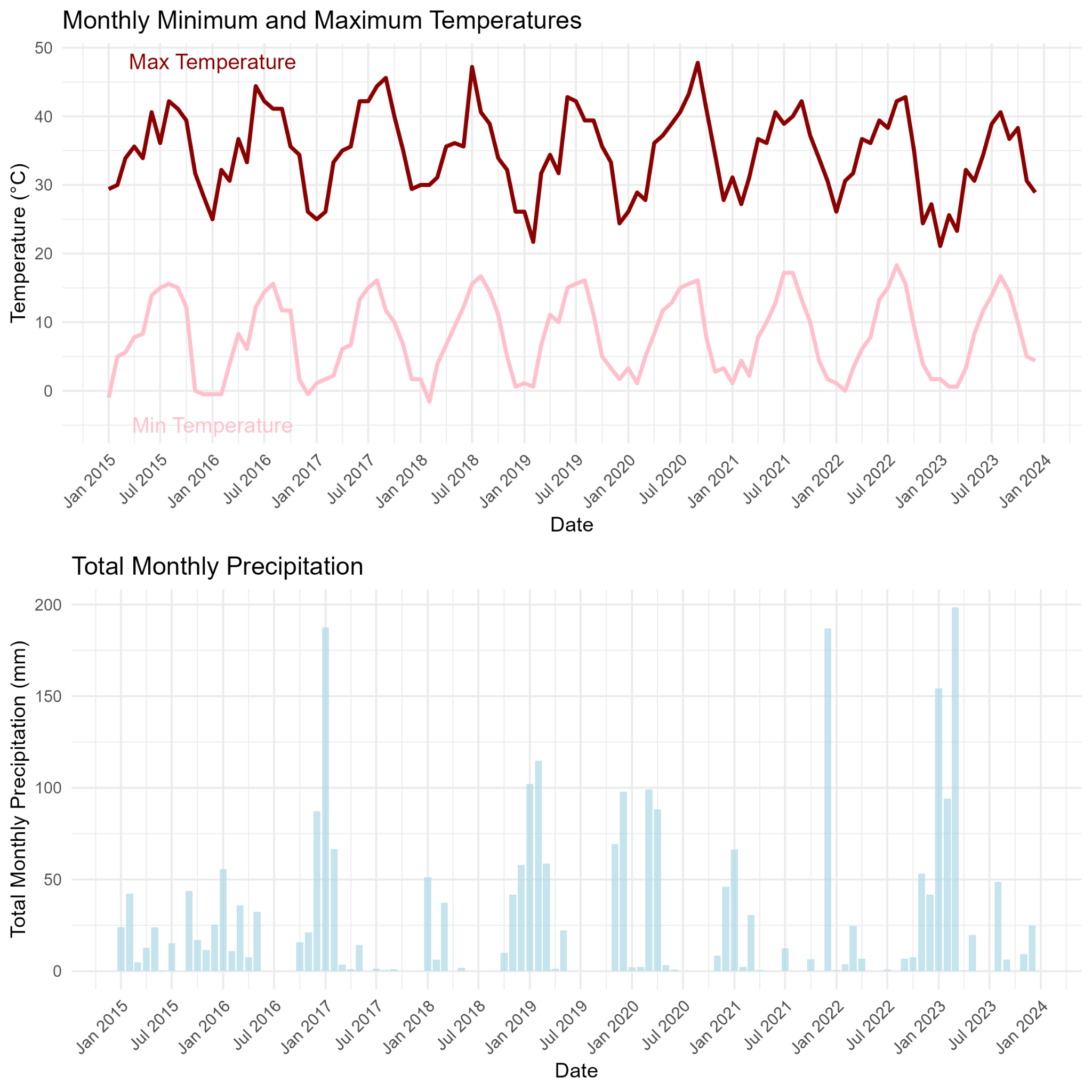


**Figure S2.** **General meteorological patterns by month during the study period**. The top panel indicates mean maximum and minimum temperatures for each month during the 2015 - 2023 study period. The middle panel indicates the mean total precipitation by month during the study period. The lower panel indicates the mean number of adult Ae. *aegypti* captured per trap across the study time period (see Table 3 for details).


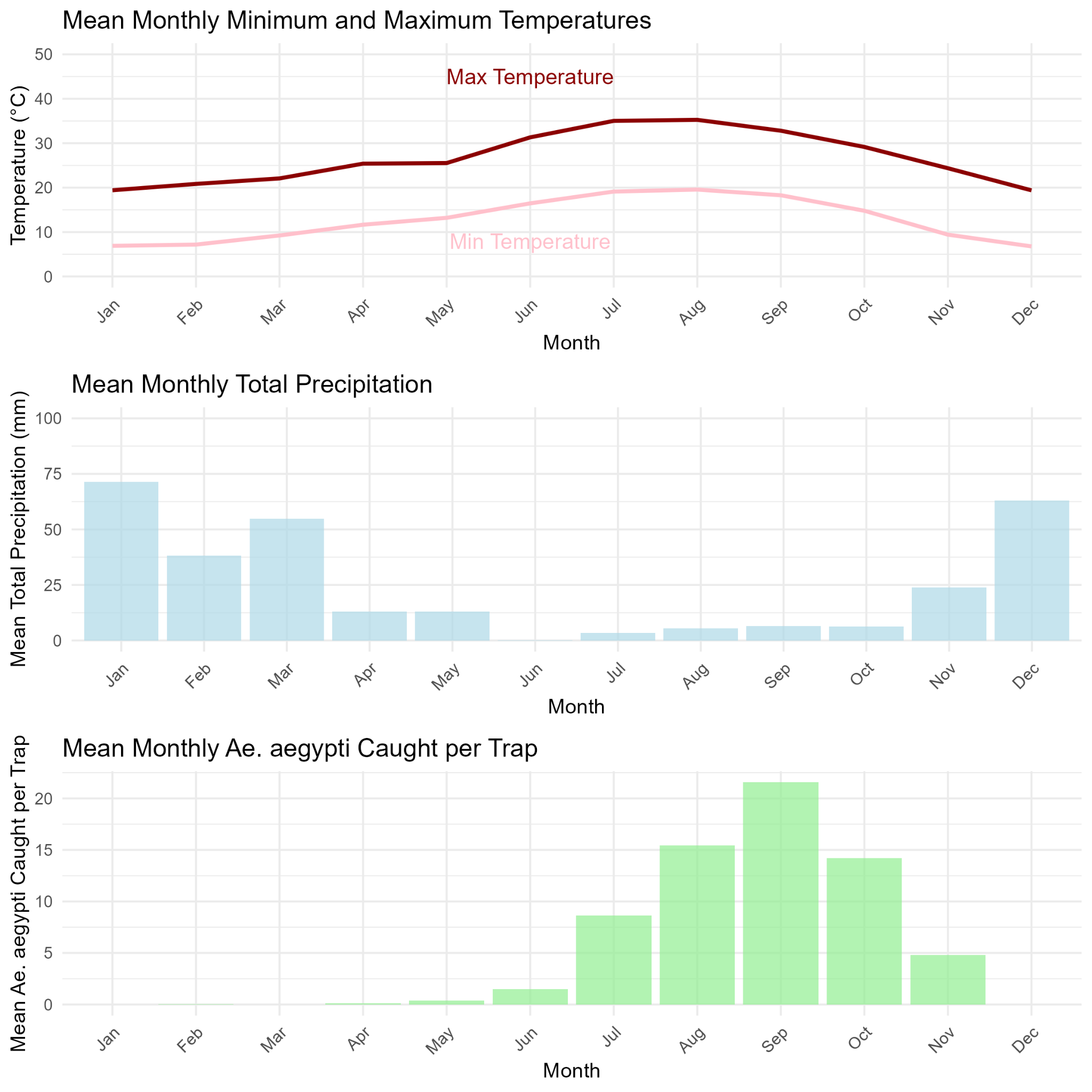


**Table S1. Summary statistics for trapped adult *Ae. aegypti* and traps by month (pooled across years).**

| **Month** | **total *Ae. aegypti* caught** | **mean per trap** | **sd per trap** | **median per trap** | **IQR per trap** | **total number of traps** | **proportion of traps with no mosquitoes** |
| --- | --- | --- | --- | --- | --- | --- | --- |
| 01 | 0 | 0.00 | 0.00 | 0 | 0 | 10 | 1.00 |
| 02 | 3 | 0.03 | 0.16 | 0 | 0 | 111 | 0.97 |
| 03 | 1 | 0.00 | 0.06 | 0 | 0 | 266 | 1.00 |
| 04 | 61 | 0.13 | 0.70 | 0 | 0 | 484 | 0.93 |
| 05 | 283 | 0.37 | 1.14 | 0 | 0 | 755 | 0.82 |
| 06 | 1277 | 1.48 | 4.12 | 0 | 2 | 860 | 0.58 |
| 07 | 7185 | 8.63 | 38.35 | 3 | 7 | 833 | 0.30 |
| 08 | 18528 | 15.44 | 21.79 | 8 | 18 | 1200 | 0.18 |
| 09 | 23937 | 21.56 | 35.12 | 11 | 24 | 1110 | 0.17 |
| 10 | 13287 | 14.21 | 23.79 | 6 | 16 | 935 | 0.18 |
| 11 | 1071 | 4.80 | 13.71 | 1 | 3 | 223 | 0.45 |
| 12 | 0 | 0.00 | 0.00 | 0 | 0 | 44 | 1.00 |

**Table S2. Summary table of Generalized Additive Model with a Negative Binomial Distribution for 14 days lags.**

| Smooth Term | edf | Ref.df | Chi.sq | p-value |
| --- | --- | --- | --- | --- |
| Longitude*Latitude | 73.14 | 99 | 776.229 | <0.001 |
| Surface water | 7.55 | 9 | 74.584 | <0.001 |
| Elevation | 0.0013 | 4 | 0.000 | 0.6701 |
| Urban area | 0.0026 | 9 | 0.002 | 0.4255 |
| Precipitation | 0.709 | 9 | 2.274 | 0.0724 |
| Temperature | 5.59 | 9 | 51.967 | <0.001 |
| DOY (Day of Year) | 6.61 | 9 | 1543.317 | <0.001 |
| Year | 1.99 | 2 | 2377.992 | <0.001 |
| DOY * Temperature | 3.39 | 4 | 59.806 | <0.001 |

Deviance explained = 68.2; AIC= 30885.02

**Table S3. Summary table of Generalized Additive Model with a Negative Binomial Distribution for 28 days lags.**

| Smooth Term | edf | Ref.df | Chi.sq | p-value |
| --- | --- | --- | --- | --- |
| Longitude*Latitude | 73.00 | 99 | 789.75 | <0.001 |
| Surface water | 6.34 | 9 | 67.87 | <0.001 |
| Elevation | 0.000651 | 4 | 0.0 | 0.780 |
| Urban area | 0.000874 | 9 | 0.0 | 0.592 |
| Precipitation | 0.000999 | 9 | 0.0 | 0.962 |
| Temperature | 0.002672 | 9 | 0.0 | 0.641 |
| DOY (Day of Year) | 6.98 | 9 | 4450.37 | <0.001 |
| Year | 1.99 | 2 | 2382.63 | <0.001 |
| DOY * Temperature | 2.96 | 4 | 37.57 | <0.001 |

Deviance explained = 67.7; AIC= 30947.68

**Figure S3.** **Spline function results from the GAM (generalized additive model) for *Ae. aegypti* abundance from 2017 - 2023 with 28 days lags.**

(A) Geographic Coordinates; (B) NDWI (Normalized Difference Water Index); (C) Elevation (DEM); (D) Built Environment; (E) Precipitation; (F) Average Temperature; (G) Day of the Year (DOY, Seasonality); (H) Year; (I) Interaction between Day of Year and Average Temperature.

###


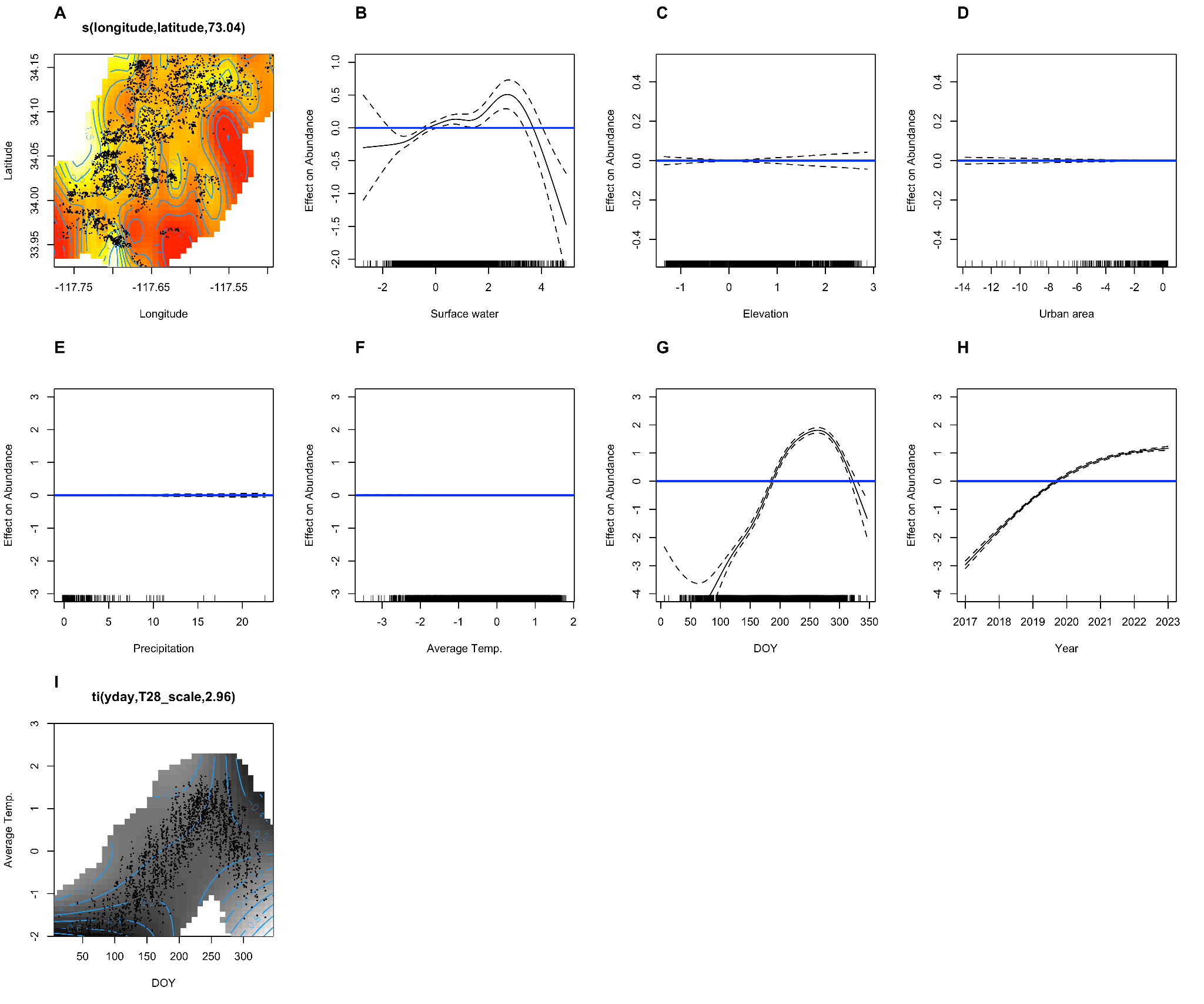


**Spatial Analysis**

**Figure S4. Spatial correlogram for *Aedes* Mosquitoes from 2017 - 2023 with 250m interval


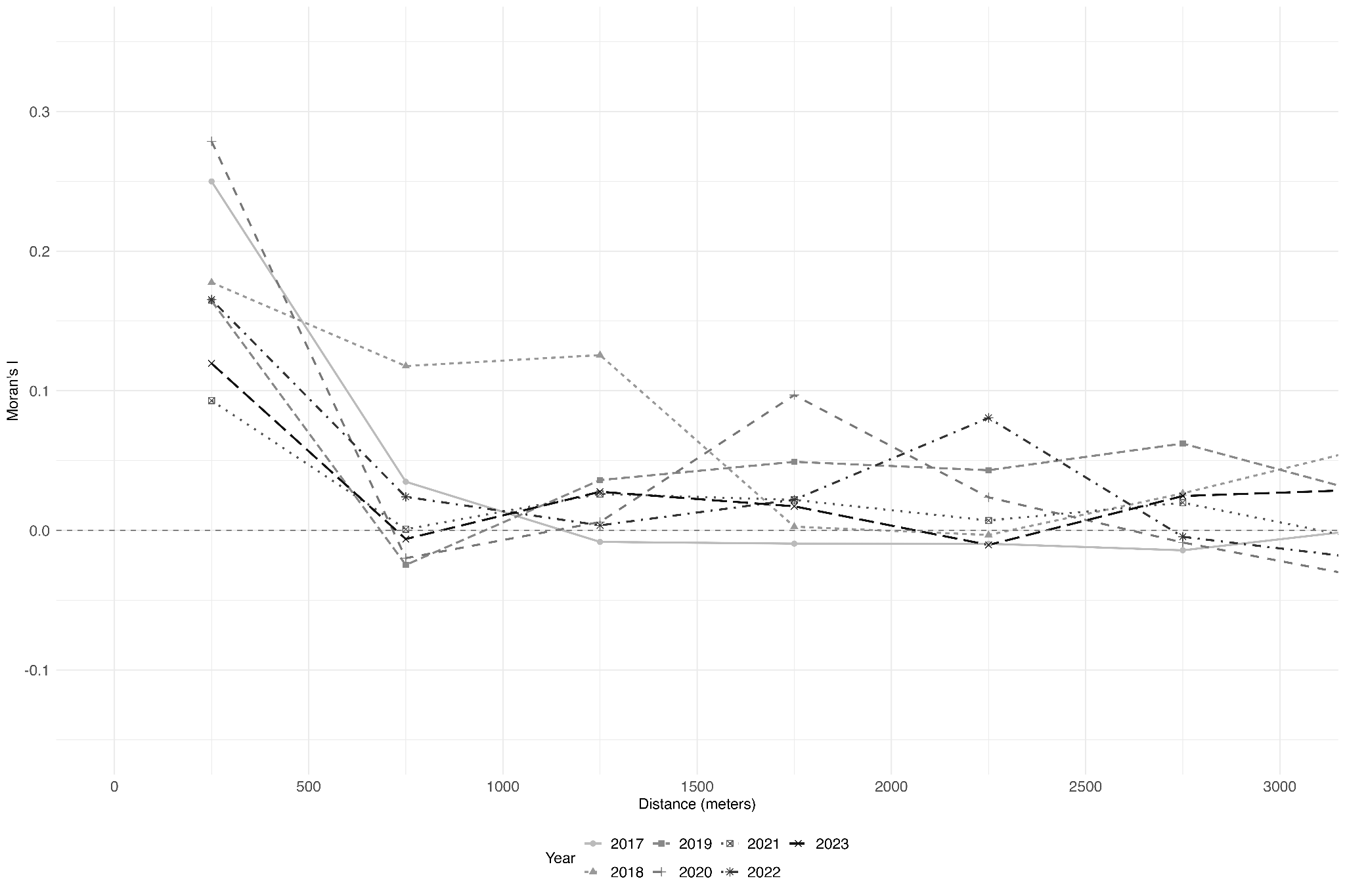
**

**Figure S5. Hotspot Analysis of *Ae. aegypti* Abundance (2017–2023) with 250m threshold.**These results show year-to-year differences in the spatial distribution of *Ae. aegypti* abundance across the study area.

**
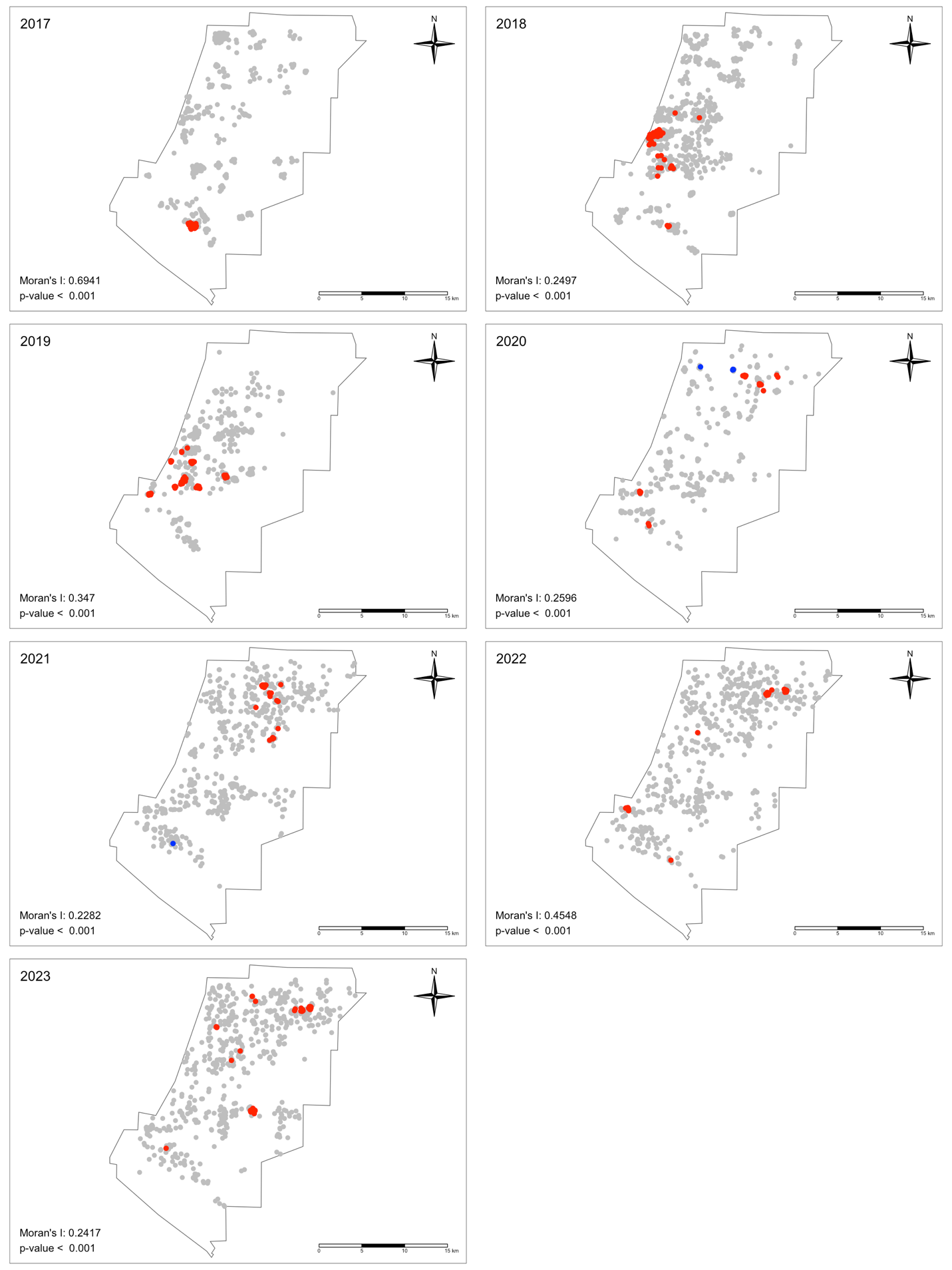
**

**Figure S6. Directed Acyclic Graph (DAG) for Mosquito Abundance in San Bernardino.**

This Directed Acyclic Graph (DAG) illustrates the complex relationships affecting mosquito abundance through various environmental and urban factors. Mosquito abundance is directly impacted by surface water, precipitation, average temperature, and elevation. Surface water, in turn, is influenced by precipitation and built area. Average temperatures are shaped by seasonal changes and elevation. Precipitation is affected by seasonal patterns, while built area is determined by elevation and the passage of time. In this DAG, surface water and precipitation act as mediators, explaining how environmental changes influence mosquito abundance. Elevation and seasons are confounders, impacting both the primary variables and mosquito abundance.


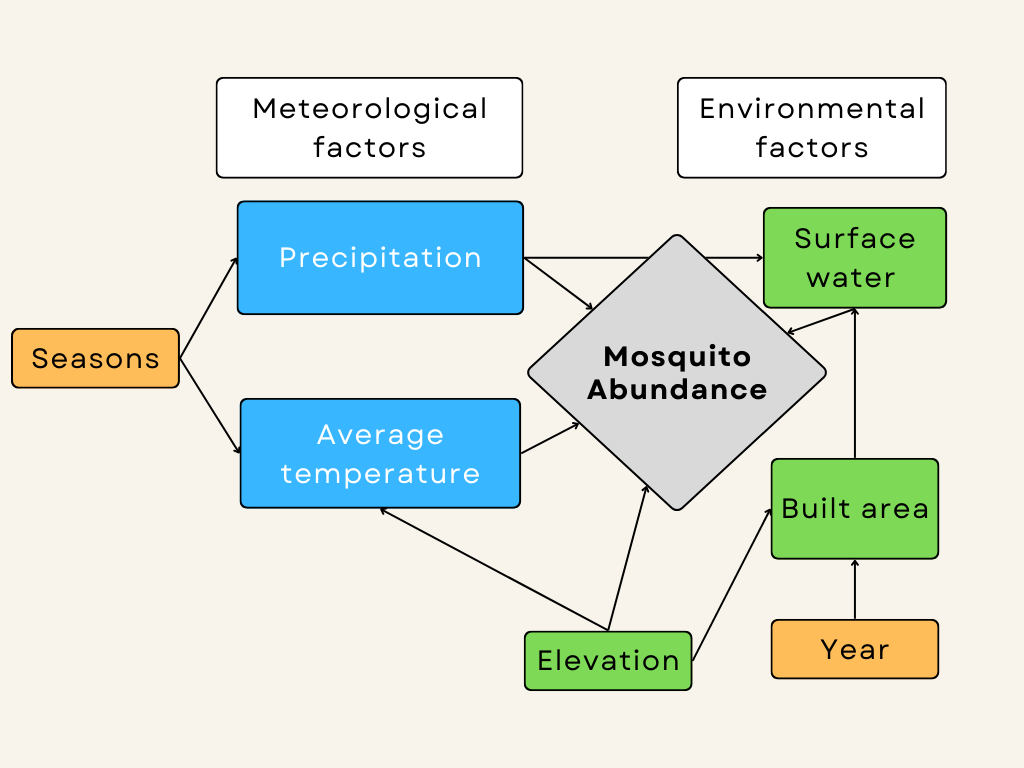


**Figure S7. Geographic Coordinates contour plot from the GAM lag 14 days.**

This is the interaction spline function for the location of traps in the main GAM. The results can be interpreted in three dimensions, and are presented here as a contour map and with colors indicating the relative values of the effect of location on *Ae. aegypti* abundance. Lighter values indicate areas on the contour plot associated with higher counts of *Ae. aegypti* mosquitoes and dark red areas indicate locations with low abundance.


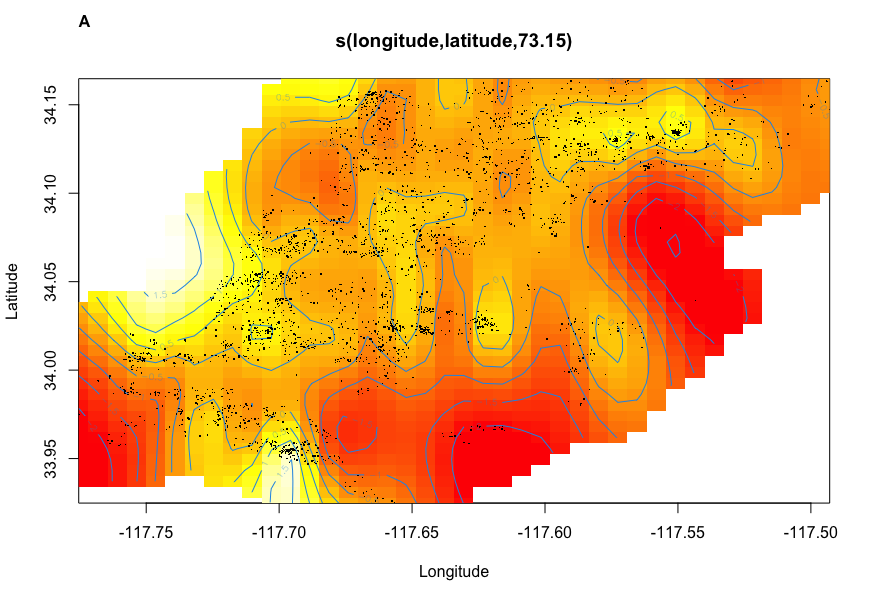


**Figure S8. Enlarge Interaction Term between Day of Year and Average Temperature from the GAM lag 14 days.**This is the interaction spline for ambient temperature (y-axis, centered on its mean) and day of year (DOY) on the x-axis from the main GAM. The results can be interpreted in three dimensions, and are presented here as a contour map and with colors indicating the relative values of the effect of location on *Ae. aegypti* abundance. Lighter values indicate areas on the contour plot associated with higher counts of *Ae. aegypti* mosquitoes


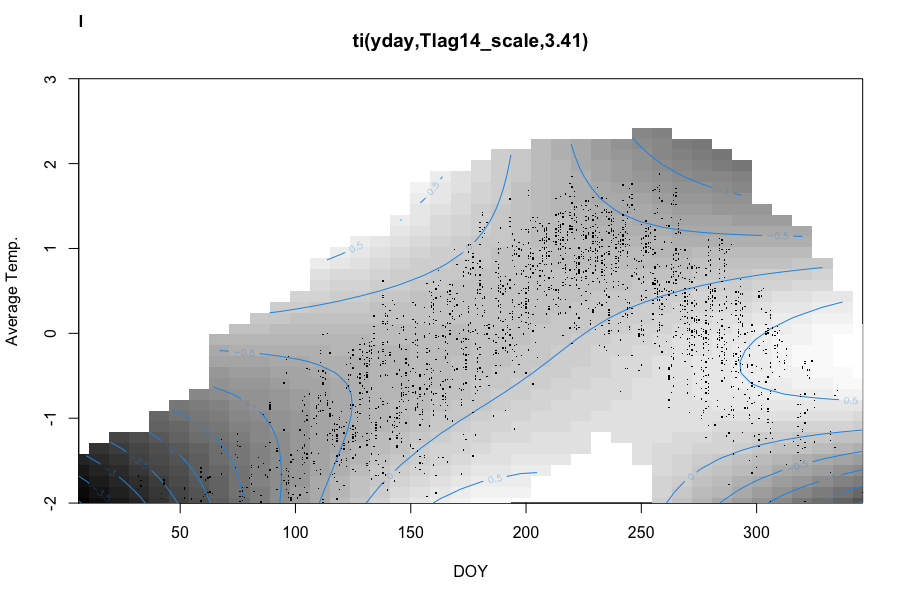


**Figure S9. Spatial Distribution of Pearson Residuals from GAM**
This is a map of Pearson residuals from the final generalized additive model (GAM), displayed at each trap location in the study area. Residual values were visualized using a custom color scale ranging from light purple (lower residuals) to orange (higher residuals), without capping. Orange shades highlight locations where the model underpredicted mosquito abundance, while lighter purple tones indicate closer model fit. The spatial distribution suggests some residual autocorrelation remains. A Moran’s I test on the Pearson residuals returned a standard deviate of 3.39 (p = 0.00034).


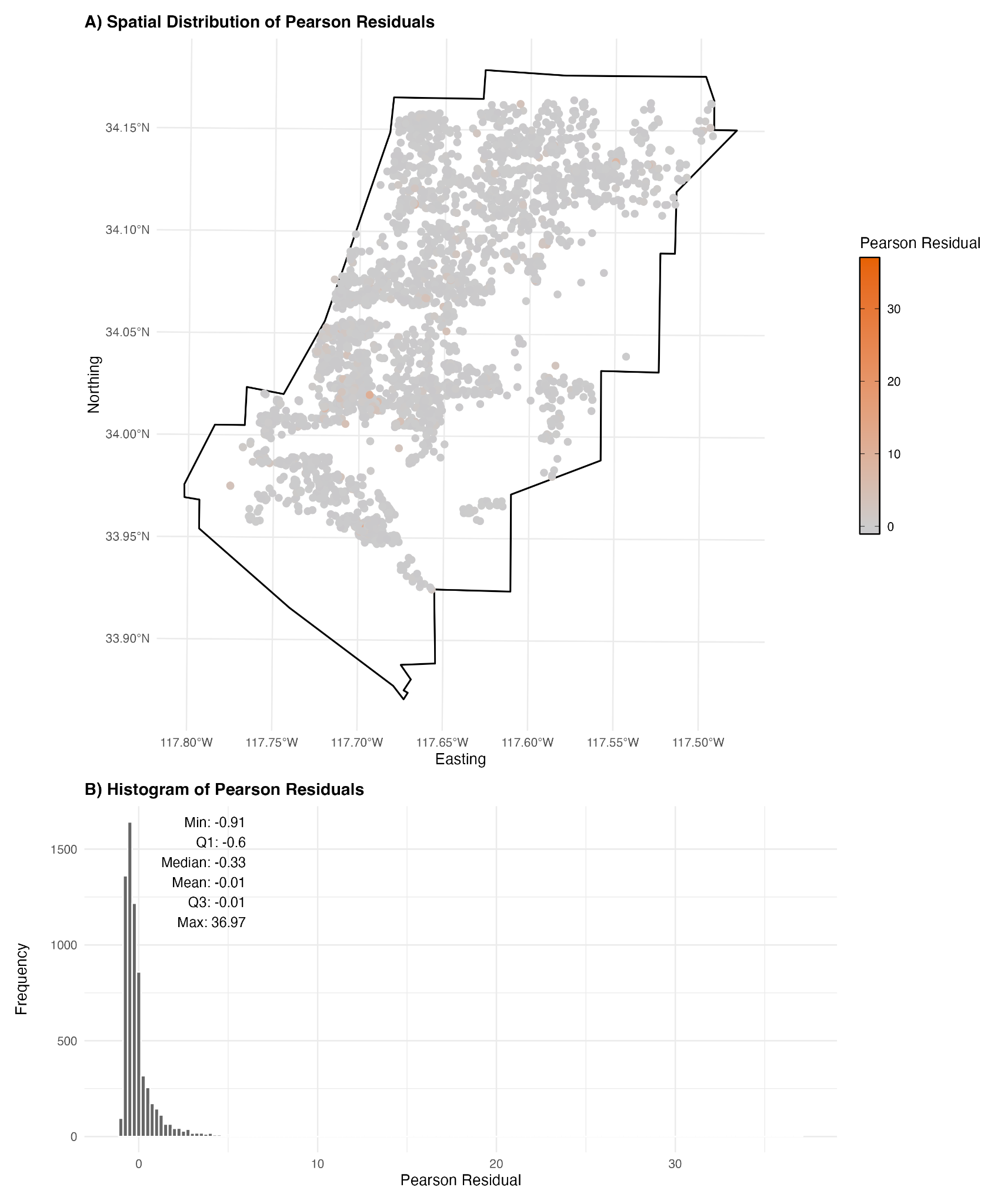


**Figure S10.** (A) Map of mosquito trap sites deployed from 2017–2023 across the study area. Red points indicate the eight routine trap sites monitored weekly throughout the study period.
(B) Monthly trends in *Ae. aegypti* abundance at each of the eight routine sites, demonstrating consistent temporal coverage over time.
